# Plant Kunitz Inhibitors and Their Interaction with Proteases: Current and Potential Pharmacological Targets

**DOI:** 10.3390/ijms23094742

**Published:** 2022-04-25

**Authors:** Camila Ramalho Bonturi, Ana Beatriz Silva Teixeira, Vitória Morais Rocha, Penélope Ferreira Valente, Juliana Rodrigues Oliveira, Clovis Macêdo Bezerra Filho, Isabel Fátima Correia Batista, Maria Luiza Vilela Oliva

**Affiliations:** 1 Departamento de Bioquímica, Universidade Federal de São Paulo, Escola Paulista de Medicina, Rua Três de Maio, 100, São Paulo 04044-020, SP, Brazil; camilabntr@gmail.com (C.R.B.); ateixeira@unifesp.br (A.B.S.T.); vm.rocha@unifesp.br (V.M.R.); penelopefvalente@gmail.com (P.F.V.); ro_juliana@yahoo.com.br (J.R.O.); clovisfilho@gmail.com (C.M.B.F.); 2 Center of Excellence in New Target Discovery (CENTD), Instituto Butantan, Av. Vital Brasil, 1500, São Paulo 05503-900, SP, Brazil

**Keywords:** cancer, coagulation, inflammation, insecticide, protease inhibitor, Kunitz, thrombosis, tumor cells

## Abstract

The action of proteases can be controlled by several mechanisms, including regulation through gene expression; post-translational modifications, such as glycosylation; zymogen activation; targeting specific compartments, such as lysosomes and mitochondria; and blocking proteolysis using endogenous inhibitors. Protease inhibitors are important molecules to be explored for the control of proteolytic processes in organisms because of their ability to act on several proteases. In this context, plants synthesize numerous proteins that contribute to protection against attacks by microorganisms (fungi and bacteria) and/or invertebrates (insects and nematodes) through the inhibition of proteases in these organisms. These proteins are widely distributed in the plant kingdom, and are present in higher concentrations in legume seeds (compared to other organs and other botanical families), motivating studies on their inhibitory effects in various organisms, including humans. In most cases, the biological roles of these proteins have been assigned based mostly on their in vitro action, as is the case with enzyme inhibitors. This review highlights the structural evolution, function, and wide variety of effects of plant Kunitz protease inhibitors, and their potential for pharmaceutical application based on their interactions with different proteases.

## 1. Introduction

Protease inhibitors (PIs) are widely distributed in nature, including in animals, plants, and microorganisms. Particularly abundant in plants, PIs account for around 1–10% of the total protein in storage organs, such as seeds and tubers [1,2]. Classified into families based on their structural features, plant inhibitors generally share cysteine patterns, overall 3D structure, and mechanism of action. The most traditional classification system sorts the inhibitors into different families, including Kunitz, Bowman–Birk, Kazal, Potato I, Potato II, and Serpins [3]. The Rawlings classification is based on similarities in the amino acid sequence, grouping the inhibitors according to their structures [4,5,6]. Consolidated aspects include molecular mass, cysteine content, and disulfide bonds; for example, members of the serine protease inhibitor family (Serpin family) exhibit well-conserved structures composed of nine α-helices and three β-sheets [7]. The Bowman–Birk family are 8–10 kDa multifunctional double-headed inhibitors (two distinct reactive sites) linked by seven disulfide bonds [8], and the Kazal family (containing at least one Kazal inhibitory domain) is widespread among microorganisms, invertebrates, vertebrates, and plants, and is characterized by six highly conserved cysteine residues forming three intradomain disulfide bonds [9].

Another important family of protease inhibitors is the plant Kunitz-type, which has a molecular weight of 20–22 kDa, and may be composed of one or two polypeptide chains linked by one or two disulfide bonds [6]. Their structures may lack cysteine residues [10,11] or contain up to five Cys residues.

Along with the study of the structural and kinetic aspects of protease inhibitors, the analysis of their interaction with proteolytic enzymes has contributed to the understanding of the phenomena involved in the control of proteolysis (Graphical Abstract). Systematic studies with isolated plant inhibitors have shown that structurally characterized proteins can be used as useful models, especially to study the variability of enzyme specificity, helping us understand the mechanism of cellular action of proteases in pathophysiological phenomena and the development of microorganisms. 

## 2. Plant Kunitz Inhibitors

Although widely distributed in nature, plant Kunitz inhibitors (I3) are especially abundant in the Fabaceae (Leguminosae) family, which comprises the Mimosoideae, Caesalpinioideae, and Faboideae/Papilionoideae subfamilies [10]. The Soybean Kunitz trypsin inhibitor (SKTI) was the first member of this family to be identified [12], and was reported by M. Kunitz; inhibitors subsequently discovered that share similar characteristics are therefore named Kunitz inhibitors. Generally, Kunitz inhibitors are defense proteins, which exhibit different activities as highlighted in this review, including antibacterial and antifungal activities, and act on inflammation, coagulation, thrombosis, and cancer.

**Figure 1:**
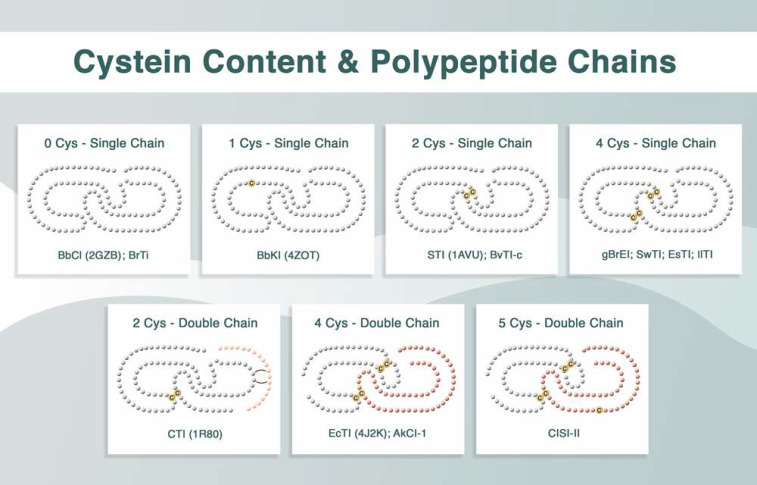
Structural aspects of plant Kunitz inhibitors in terms of the number of cysteine residues (0, 1, 2, 4, or 5 Cys), S–S bridges (none, intrachain and/or interchain bridges), and polypeptide chain representation (single or double chains).

Plant Kunitz inhibitors generally exhibit highly conserved primary structures. These inhibitors usually have a reactive site located in the region that binds to the enzyme, and the formation of the enzyme–inhibitor complex occurs in 1:1 stoichiometry. Their reactive sites are frequently composed of Arg and Lys; however, they may occasionally contain Glu, Ala, or Val [6]. These inhibitors can be single- or double-polypeptide-chain proteins, with variation in the number of cysteine residues and disulfide bridge patterns. The *Bauhinia bauhinioides* cruzipain inhibitor (BbCI) [13] and *Bauhinia rufa* trypsin inhibitor (BrTI) [14] are single-chain inhibitors lacking cysteine residues. The *B. bauhinioides* kallikrein inhibitor (BbKI) is also a single-chain inhibitor; however, it contains one cysteine residue [11,15]. The *B. rufa* elastase inhibitor (gBrEI) [16], *Swartzia pickellii* trypsin inhibitor (SWTI) [17], *Entada scandens* trypsin inhibitor (ESTI) [18], and *Inga laurina* trypsin inhibitor (IlTI) [19] are single-chain inhibitors with two cysteine residues. Although the *Copaifera langsdorffii* inhibitor (ClI) also contains two cysteines, it is a double-chain inhibitor, with the chains linked through noncovalent bridges [20]. The classic Kunitz-type inhibitors, soybean trypsin inhibitor (STI) [21], and *Bauhinia variegata* var. candida trypsin inhibitor (BvTI-C) [22] are single-polypeptide-chain proteins that possess four cysteines. The *Enterolobium contortisiliquum* trypsin inhibitor (EcTI) [23,24] and *Acacia karroo* trypsin inhibitor (AkTI) [25] also contain four cysteines; however, they are double-polypeptide-chain proteins linked by two S–S bridges. *Canavalia lineata* subtilisin inhibitor type II (CLSI-II) is a similar double-chain inhibitor linked by two S–S bridges, but contains five Cys residues, four of which are linked with bridges and one is a free cysteine [26]. These profiles are summarized in Figure 1. 

## 3. Recombinant Kunitz Inhibitors

Natural compounds such as plant proteins have been used for a long time to improve human health. However, the yield of protease inhibitors obtained from natural plant sources is generally low. With the advent of genetic engineering, it is now possible to obtain large amounts of these inhibitors as recombinant proteins at a low cost, while maintaining or even improving their inhibitory activities.

The recombinant *B. bauhinioides* kallikrein inhibitor (rBbKI) and recombinant *B. bauhinioides* cruzipain inhibitor (rBbCI) were obtained by heterologous expression and production in *Escherichia coli* [27]. While BbCI (a cruzipain and elastase inhibitor) and BbKI (effective in inhibiting coagulation and fibrinolysis enzymes) share 82% sequence similarity, their reactive sites show slight differences. Based on this information, rBbKI was constructed by mutating the R64 residue in the BbKI reactive site to A64, as found in BbCI. This single mutation modified the specificity of BbKI, turning it into a weak plasma kallikrein inhibitor (K_iapp_ = 2.0–98 nM) and a strong plasmin inhibitor (K_iapp_ = 33–2.6 nM) [11]. Thus, the recombinant inhibitor can prevent fibrin breakup and maintain blood clot stability, which opens up new avenues for its application as a therapeutic protein (Table 1).

The same research group obtained rBbKIm based on the primary sequence of BbKI, but incorporated the RGD/RGE (Arg–Gly–Asp/Arg–Gly–Glu) motifs from BrTI. To this end, the mutations V21E, S24A, H25R, H27D, A28G, E127D, and Q130E were introduced into BbKI. The importance of these residues in the defense against predatory insects has been demonstrated [28]. This inhibitor also interfered with the viability of prostate cancer DU145 and PC3 cells, leading to cell death via the release of cytochrome c and caspase-3 activation [29] (Table 2).

Moreover, the *Ligusticum chuanxiong* subtilisin/alpha-amylase inhibitor (LASI), expressed in *E. coli* Rosetta and obtained from the rhizome of *L. chuanxiong* (frequently used in Chinese medicine to treat headaches, cardiovascular diseases, rheumatic joint pain, menstrual disorders, and painful swelling), was also obtained as a recombinant protein. LASI inhibits mammalian alpha-amylase and bacterial subtilisin (which may be involved in the protection of plants against microorganisms) and also affects pest lepidopterans, which are all relevant for agricultural applications. Although LASI shares sequence similarities with other Kunitz protease inhibitors, such as *Cynara cardunculus* (68%), it has a unique reactive site; consequently, LASI inhibits subtilisin but does not affect trypsin or chymotrypsin. Furthermore, LASI inhibits alpha-amylase from *Plutella xylostella*, *Helicoverpa armigera*, and *Spodoptera litura*, thereby reducing the survival of these pests [30].

The insecticidal properties of the *Cassia obtusifolia* trypsin inhibitor (CoTI1) have also been described [31]. CoTI1 was used to investigate stress tolerance and increase the resistance of crops against pests. It is a potent trypsin inhibitor (2.490 UI/mg activity) and also inhibits trypsin-like proteases from *H. armigera*, *S. litura*, and *S. exigua* (1.770 UI/mg, 1.510 UI/mg, and 1.210 UI/mg, respectively). The amino acids L84, R86, and T88 in CoTI1 were identified, using in silico analysis, as being involved in trypsin inhibition, which was further confirmed by the loss of inhibitory activity (54%, 90%, and 53%, respectively) on mutating these residues to alanine.

Inhibition of human neutrophil elastase (HNE) is known to be beneficial in modulating inflammatory lung diseases. The *Caesalpinia echinata* elastase inhibitor (CeEI), which inhibits HNE, has been used to study pulmonary inflammation [32]. Native CeEI is a potent inhibitor of HNE (K_i_ 1.9 nM), cathepsin G (CatG, K_i_ 3.6 nM), and protease 3 (K_i_ 3.7 μM). Two recombinant isoinhibitors of CeEI, rCeEI-4, and rCeEI-5 were cloned and expressed in *E. coli*. rCeEI-5 inhibited HNE 6.2 times more efficiently than CeEI, while rCeEI-4 exhibited 4.2-fold higher inhibition of chymotrypsin and 5.3-fold higher inhibition of HNE than CeCI. Synthetic, smaller functional peptides that inhibit HNE rCeEI-36 (K_i_ = 0.3 nM) and rCeEI-46 (K_i_ = 8.8 nM) were also obtained from the CeEI-4 sequence.

*Apios americana*, a legume tuber plant widely distributed across North America, is commonly used to make bread in Japan [33]. This plant produces two Kunitz-type protease inhibitors, *Apios americana* protease inhibitor 1 (AKPI-1) and *Apios americana* protease inhibitor 2 (AKPI-2), which were cloned and expressed as recombinant proteins. rAKPI-2, characterized as chymotrypsin (K_i_ 6 µM) and trypsin inhibitor (K_i_ 0.4 µM), loses its property of trypsin inhibition when heated at 70 °C; however, the chymotrypsin inhibitory activity is maintained at 80% of its initial value at the same temperature.

Although most recombinant inhibitors are produced in bacterial hosts, it is also possible to do so with yeast, as achieved by Bunyatang et al. [34]. The PKPI protease inhibitor, a bifunctional inhibitor that acts on protease and amylase, was cloned and transferred into *Pichia pastoris* to produce a full-length protease inhibitor based on the *Hevea brasiliensis* inhibitor (HbASI), which showed high activity against alpha-amylase (from *Aspergillus oryzae*, IC_50_ 200 µg), subtilisin A (IC_50_ 380 µg), and trypsin (IC_50_ 325 µg), but no activity against chymotrypsin and human alpha-amylase. In addition, HbASI showed good stability over a wide temperature and pH range, and inhibited mycelial growth of *Phytophthora palmivora* (0.2 µM), including 50% inhibition of zoospore (Table 2).

## 4. Defense Proteins

Plants can detect events locally and transmit defense signals to build up systemic acquired resistance (SAR). A Kunitz protease inhibitor in the tea plant *Camellia sinensis* (CsKPI1) was shown to be potentially involved in SAR based on its gene expression, which was strongly induced by insects through localized feeding, and in neighboring undamaged leaves. Jasmonic acid and CsKPI1 were detected simultaneously in damaged leaves; in addition, the jasmonic acid content also increased in neighboring leaves, suggesting its role as a vital hormonal signal that induces the expression of CsKPI1 in SAR [67].

**Figure 2:**
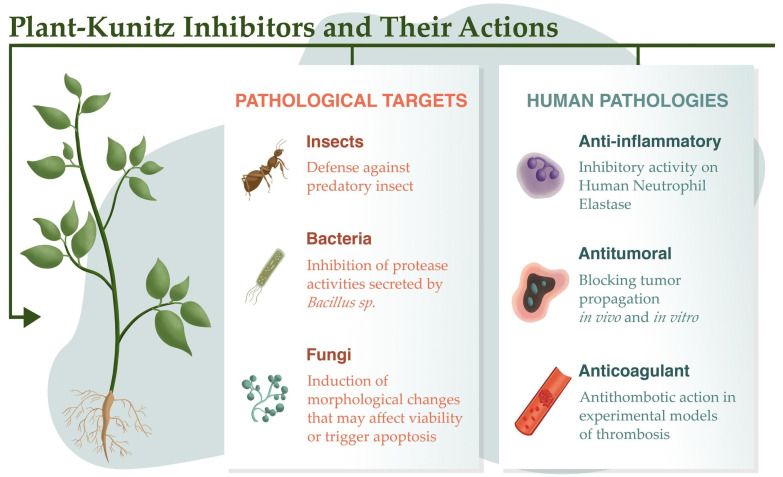
Uses of protease inhibitors as insecticides, and antibacterial and antifungal agents. The inhibitors are also active in mammal pathologies associated with inflammation, thrombosis, and tumorigenesis.

Based on their ability to function as defense proteins that protect against predator attacks, protease inhibitors have been used as insecticidal agents that act by inhibiting digestive proteases in insects, thereby controlling the availability of amino acids for the growth and development of larvae (Figure 2, Table 2). The Kunitz-type *Cassia leiandra* trypsin inhibitor (ClTI) inhibits midgut digestive proteases (50% activity reduction at 4.65 µM) and larvae development (after 10 days at a final concentration of 15.4 µM), and delays adult emergence, in *Aedes aegypti*, causing 44% mortality. These findings suggest that ClTI has the potential to reduce the *A. aegypti* population without increasing the risk of developing insect resistance [43]. Similarly, alocasin, purified from the rhizome of *Alocasia macrorrhiza*, increased the mortality rate of *A. aegypti* larvae and adult females (IC_50_ = 0.17 mg/mL) by its action against the midgut enzymes of this insect. However, higher inhibitor concentrations lead to a reduction in the inhibitory effect, which may be attributed to the production of insect proteases insensitive to the inhibitor upon prolonged exposure [38]. In another study, proteolytic activity was restored on the 15th day after hatching of *Anticarsia gemmatalis* larvae exposed to SKTI, after a decrease in the total proteolytic activity of trypsin and similar enzymes until the 12th day [68]. On the other hand, the *Adenanthera pavonina* Kunitz-type inhibitor (ApKTI) acts through a noncompetitive mechanism, thereby offering an advantage over such adaptation mechanisms as its affinity for trypsin is not affected by amino acid mutations in the enzyme active site, unlike other competitive protease inhibitors. Thus, ApKTI showed insecticidal activity even after 15 days of chronic exposure when included in the artificial diet of *Plodia interpunctella* larvae [36], and achieved a 60% reduction in the survival of neonatal *Anticarsia gemmatalis* larvae [37]. According to Sasaki et al. [69], *A. aegypti* larvae also do not use regulatory mechanisms to overcome the effects of ApKTI, and the inhibitor demonstrated degeneration of the microvilli of epithelial cells of the posterior midgut region, hypertrophy of the gastric cecal cells, and an augmented ectoperitrophic space in larvae. Interestingly, although trypsin and chymotrypsin-like serine proteases are involved in the initial protein digestion of Lepidoptera, ILTI (from *Inga laurina*) inhibits trypsin in the midgut of *S. frugiperda* more effectively compared to other protease inhibitors, such as SKTI and *Inga vera* trypsin inhibitor (IVTI). Neither SKTI nor ILTI was active against chymotrypsin. Notably, ILTI was not degraded by enzymes in the midgut of insects, and was stable after elimination in feces, showing resistance to microbial metabolism [59]. IVTI exerted midgut inhibition in *S. frugiperda*, *C.* *cephalonica*, *H. virescens*, and *H. zea* of 83%, 80%, 70%, and 64%, respectively [60]. More recently, other inhibitors have been characterized, such as RsKI from *Rhynchosia sublobata*, which is active against lepidopteran gut proteases, including those of *Achaea janata* (IC_50_ = 1.95 µg) and *Helicoverpa armigera* (IC_50_ = 59 µg) [66], and the inhibitor CpPI 63, isolated from *Cajanus platycarpus* seeds, a wild relative of pigeonpea [70].

While protease inhibitors are important in defending against predators, they can also selectively assist in mutualistic relationships. For example, the ant *Pseudomyrmex ferrugineus* uses *Acacia* sp. hollow thorns as a nesting space, and while the *Acacia* sp. provides food bodies and extrafloral nectar for ant larvae, the ant offers protection against herbivores that compete for vegetation. Thus, protease inhibitors extracted from *Acacia* sp. food bodies are activated and convert the nutritive food reward into something difficult to digest for potential or optional exploiters, but not for mutualistic consumers, whose biochemistry takes advantage of this reward [71].

Cowpeas are an important food source for millions of people worldwide. Many studies have used *Callosobruchus maculatus* [72] and *Zabrotes subfasciatus* [73] as models to evaluate the effect of protease inhibitors because these beetles cause serious damage to the grain, making it unfit for human consumption. The *Pithecellobium dumosum* Kunitz inhibitor (PdKI), purified from the seeds of the *P. dumosum* tree, was evaluated against cysteine and serine proteases extracted from the larvae of coleopterans *C. maculatus* and *Z. subfasciatus* and the lepidopterans *Alabama argillacea* and *Telchin licus*. It completely inhibited bovine trypsin at 100 nM, with 50% inhibition against bovine chymotrypsin. PdKI showed specificity for serine proteases, especially against those from phytophagous insects, such as Coleoptera and Lepidoptera [74]. 

Many factors are involved in the adaptability of insects to protease inhibitors, such as the alternative use of other classes of proteases, overexpression of proteases to minimize the inhibitory effect, or the degradation of the inhibitors by nontarget proteases, which are insensitive to the inhibitors. Thus, despite the potential applicability of protease inhibitors as effective compounds for the protection of crops against herbivory, most recent studies have pointed to the importance of multitrophic approaches, taking into account not only target insect proteases, but also proteases from other organisms in the food chain, including the plant itself [75].

Termites are one of the main wood-destroying pests that can invade and attack the wooden building structures in urban environments. While synthetic pesticides are traditionally used to prevent termite attacks, these compounds tend to harm humans and the environment. As an alternative, the trypsin inhibitor isolated from *Cassia grandis* seeds (CgTI) can be employed, which exhibits termiticidal activity against *Nasutitermes corniger*. However, the activity has been found to vary between termite castes, with workers (IC_50_ = 0.685 mg/mL) being more resistant than soldiers (IC_50_ = 0.765 mg/mL) [42], which can be attributed to variation in enzymatic activities and digestive tracts between the castes. Similar to these results, EcTI was able to induce the death of worker termites, with an IC_50_ of 0.242 mg/mL, whereas it did not exert significant inhibition in soldier termites. Notably, the molecule from the intestinal extract that binds to EcTI is a chitinase from *T. reesei*, a symbiotic fungus present in the digestive tract of termites that is important in the cellulose degradation process. As this cellulose is exploited for the production of bioethanol, EcTI could be applied as a biotechnological tool in addition to being a natural active termiticide [50]. 

In the same line of investigation, two distinct recombinant protease inhibitors of *B. bauhinioides* origin, rBbKI (serine protease inhibitor) and rBbCI (cysteine protease inhibitor) were evaluated for their termiticidal activity. Despite sharing 82% structural similarity, the two inhibitors showed differing specificity for termite intestinal enzymes, and could be interesting tools for the characterization of *N. corniger* enzymes. rBbKI showed termiticidal activity in workers, with an LC_50_ of 0.9 mg/mL after four days, and did not affect the survival of soldiers, whereas rBbCI did not show any termiticidal activity on *N. corniger* [64]. In another study, *Araucaria angustifolia* pine nut extract was shown to exhibit termiticidal activity against *N. corniger* workers and soldiers at all tested concentrations [76]. 

Finally, the bifunctional lectin and Kunitz inhibitor AEL, isolated from *Abelmoschus esculentus*, was shown to exhibit activity against the fruit fly *Ceratitis capitata* and the nematodes *Meloidogyne incognita* and *Meloidogyne javanica*, which infest plant roots. In the pupal stage of *M. incognita*, 2 mg/mL AEL showed higher toxicity than organophosphates commonly used in agriculture, exemplifying the potential of protease inhibitors as promising insecticidal molecules [35] (Table 2).

## 5. Antibacterial and Antifungal Activities 

Fungi and bacteria release proteases into the media during infections, which facilitates their defense and survival. Inhibitors of these proteases can therefore hinder pathogen metabolism to improve human health [77]. Plant inhibitors from the Kunitz family may be important in controlling fungal and bacterial growth, as demonstrated by the inhibition of proteases secreted by *Bacillus* sp. (Figure 2) and *Aspergillus flavus* induced by extracts from seeds of *Cassia tora* (L.) *syn Senna tora* (L.) *Roxb* [78]. In addition, the isolated protein fraction from *Erythrina poeppigiana* interferes with the germination of *A. flavus* spores [79]. ClTI from *C. leiandra* seeds inhibited the growth of *Candida albicans*, *Candida tropicalis*, *Candida parapsilosis*, and *Candida krusei*, with values of 5.13 μM, being most potent for *C. albicans*, with an IC_50_ of 2.10 μM [80].

Some inhibitors show more restricted specificity, such as the *Inga edulis* trypsin inhibitor (IeTI). Although it has no action against *C. albicans*, it is a potent inhibitor of *C. tropicalis*, as well as *Candida buinensis* (87% inhibition with 400 μg/mL IeTI). The antifungal activity was found to be mediated by the alteration of the plasma membrane, which diverts energy metabolism from physiological functions to repair it, thereby inducing morphological changes that can affect cell viability or even trigger apoptosis through complexation with vital enzymes in this pathway (Figure 2) [58]. The same mechanism was described in studies with *Enterolobium timbouva* trypsin inhibitor (EtTI), suggesting that this inhibitor affects the integrity of the plasma membrane of yeasts, inducing the generation of intracellular ROS due to mitochondrial activation to maintain membrane potential, and consequently affecting the organelle structure. In addition, ROS generation is the main cause of DNA damage in yeast, and the mode of action of EtTI appears to involve the activation of apoptosis via a different pathway than traditional antifungal drugs [56,57]. This mechanism is also similar to that used by *Pseudostellaria heterophylla* trypsin inhibitor (PhTI), a 20.5 kDa thermostable inhibitor that has been reported to show potent activity against the pathogenic fungi *Candida gloeosporioides* and *Fusarium oxysporum*. The inhibitor also exhibits high stability, suggesting its potential for use as an antifungal protein in the food industry and agriculture [63].

## 6. Inflammation

Microbial invasion triggers characteristic inflammatory reactions in the host, such as the continuous migration and degranulation of neutrophils, which release host proteases, such as HNE and CatG [81]. These enzymes are important in the inflammatory response; CatG induces monocyte and neutrophil chemotaxis and activates chemokines [82], while HNE activates the epidermal growth factor (EGF) receptor and induces the expression of IL-8 [83], for example, in emphysema. Although the role of proteases in emphysema is widely accepted, it remains unclear which cells and/or proteases have key functions in disease development and progression. Serine, cysteine, and/or metalloproteinase classes have been reported as the proteases most likely to be involved in the pathogenesis of chronic obstructive pulmonary disease (COPD) and may be the reason for the successful employment of protease inhibitors, as reported for the inhibitors applied in experimental in vivo models of elastase-induced lung injury [84,85] and an asthma model [86], with a reduction in the expression of TNF-α, MMP-9, MMP-12, TIMP-1, eNOS, and iNOS cells in the airways and alveolar walls with inhibitor treatment. However, a decrease in the volume ratio of 8-iso-PGF2, collagen, and elastic fibers in the airways and alveolar walls indicates that these proteins reduce pulmonary inflammation, remodeling, oxidative stress, and mechanical changes. 

*Crataeva tapia* bark lectin (CrataBL) is another inhibitor used in this experimental model [45,46]. The inhibitor reduced the constriction of the airways and lung parenchyma. The effect was associated with a significant reduction in exhaled nitric oxide, and led to the control of the inflammatory response, with a decrease in bronchoalveolar lavage; the number of neutrophils, lymphocytes, and eosinophils; and the mean alveolar diameter. CrataBL was also able to decrease the number of TNF-α positive cells in the lung parenchyma and pulmonary airways, and macrophages in the lung parenchyma. All these investigations suggest that protease inhibitors may have therapeutic potential for the treatment of COPD and provide a better understanding of the involvement of proteases in this pathology.

Bauhinia inhibitors also exhibit protective effects on gastric mucosa [39]. The authors evaluated the in vivo effects of three Kunitz inhibitors on the prevention of gastric mucosal damage: *Bauhinia mollis* elastase inhibitor (BmEI), *B. mollis* trypsin inhibitor (BmTI), and *B. bauhinioides* cruzipain inhibitor (BbCI). These effects were compared with those of ranitidine and saline solution (vehicle). The fraction exhibiting inhibitory activity against HNE (K_iapp_ 2.8 nM) (Figure 2) and CatG (K_iapp_ 1.0 nM) exerted a significant antiulcer effect at 3 mg/kg. The same protective effect was observed after pretreatment with the BbCI inhibitor, which inhibits HNE (K_iapp_ 2.6 nM), CatG (K_iapp_ 160.0 nM), and the cysteine protease cathepsin L (K_iapp_ 0.2 nM). In rats, the administration of both BmEI and BbCI resulted in antiulcer effects, indicating the likely inhibition of elastase by both inhibitors, and confirming the relevance of elastase inhibition associated with the action of neutrophil proteases in the regulation of proinflammatory processes and stress-induced gastric mucosal injuries (Table 2).

## 7. Coagulation and Thrombosis

The activation of the blood coagulation cascade, essentially mediated by serine proteases with specificity for arginine residues, was one of the first biological events in which the importance of reactions involving proteolysis was shown. Arterial and venous thrombosis, consequences of dysfunction of this system, constitute the final triggering process of complications and mortality, not only from diseases of the cardio–circulatory system, but also from other systemic conditions, such as cancer and diabetes, trauma, and even physiological events, such as pregnancy and the postpartum period [87].

Plasma kallikrein (PKa) is involved in numerous proteolytic reactions related to the heart and blood vessels, such as the intrinsic coagulation pathway, kallikrein–kinin system, fibrinolytic system, renin–angiotensin system, complement system pathways involving vascular responses of hemostasis, and pathological conditions, such as cardiovascular and vascular disease [88,89,90].

Furthermore, although PKa does not induce platelet aggregation, it can potentiate platelet aggregation with a low concentration of ADP (2 μM) by binding to platelet integrins through their KGD and KGE motifs and mediating PAR-1 cleavage [91]. The role of coagulation factors XIIa and XIa, and more recently PKa, in thrombus formation, dissociated from hemostasis, has been highlighted by many authors [88,89,90,92]. These investigations show how these proteases contribute to vascular diseases and thrombosis in the intravascular compartment, and are attractive targets for therapeutic inhibition in the treatment and/or prevention of thrombosis.

Numerous plant-derived inhibitors acting on different proteases (released by leukocytes) in the coagulation cascade have been described in more recent studies (Figure 2), such as EcTI, a multispecific inhibitor that acts on PKa, plasmin, human neutrophil elastase, and factor XIIa. It does not, however, inhibit thrombin, human plasmin, or factor Xa (FXa) [93,94].

The BbKI/rBbKI inhibitor from *Bauhinia* species, whose inhibition constants on human PKa and rat plasma kallikrein are 2.4 nM and 5.2 nM, respectively, is the only inhibitor capable of acting on tissue kallikrein (K_iapp_ 20 µM). In addition to human plasma kallikrein, BbKI inhibits plasmin. However, this inhibitor does not act on FXa, unlike the inhibitor BuXI from *B. ungulata*, which is the only inhibitor from *Bauhinia* that inhibits this enzyme (K_iapp_ 14 nM), distinguishing itself from the BvTI with which it shares a structural similarity of approximately 70%. In addition to FXa, BuXI inhibits FXIIa (K_iapp_ 74 nM), PKa (K_iapp_ 6.9 nM), and plasmin (K_iapp_ 76 nM). BvTI inhibited plasmin (K_iapp_ 2.9 nM) more efficiently than BuXI, in contrast to PKa (K_iapp_ 80 nM) and FXIIa (K_iapp_ 110 nM), and did not inhibit FXa. The differences in the reactive site are shown to be important to determine the specificity of action. For example, methionine residues at positions 59 and 67 in BuXI play an important role in the interaction with FXa, and do not interfere with trypsin complex formation [10,95]. 

**Figure 3:**
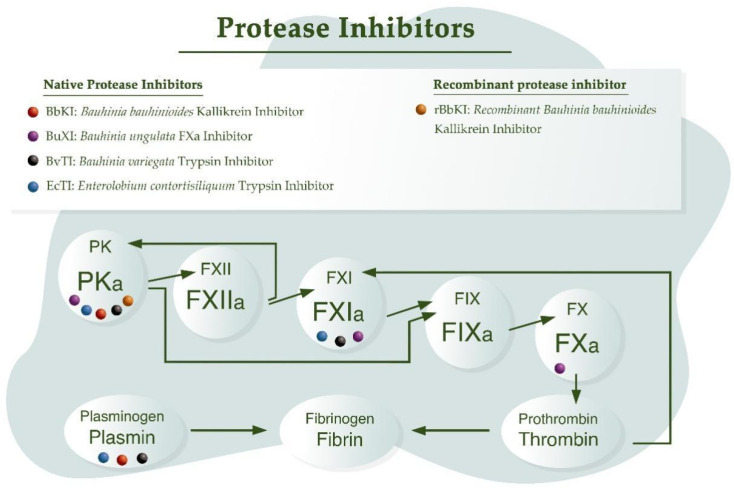
Activity of plant Kunitz inhibitors on some intrinsic pathway clotting enzymes, and on fibrinolytic enzymes. Coagulation cascade scheme based on Kearney et al. [98].

Some of these inhibitors showed antithrombotic action in different experimental models of arterial and venous thrombosis (Figure 3) [47,49,65,96,97], with little or no interference in bleeding time [47,65], suggesting a potential therapeutic value in inhibiting the development of thromboses with a lower hemorrhagic effect (Table 1). 

## 8. Cancer

In cancer, the overexpression or activation of a protease provides a proteolytically active environment that surrounds the tumor. Importantly, tumor cells overexpress these enzymes to influence the local blood supply, cell extravasation through vessels, and migration through the cell matrix during metastasis. 

The presence of protease inhibitors in legume seeds and cereals, in addition to epidemiological studies that identify legumes as possible protective agents in reducing the incidence of certain cancers in the vegetarian population, has prompted several studies to include protease inhibitors for blocking tumor propagation in vivo and in vitro (Figure 3, Table 2), although the mechanisms of inhibitor action are not fully understood [99]. Many proteins found in soybeans are being used for therapeutic purposes, one of which is a Kunitz-type protease inhibitor, with results indicating their ability to inhibit the proliferation of cancer cells. The 24.2 kDa Kunitz-type trypsin inhibitor KTI-A demonstrated antiproliferative activity (IC_50_ of approximately 6.6 µM) in the melanoma cell line, B16F1. Therefore, soybeans can be used to obtain bioactive molecules according to the requirements of pharmaceutical and other industries [62]. 

Sporamin, a Kunitz-type trypsin inhibitor found in sweet potatoes (*Ipomoea potatoes*), was shown to exert anticancer effects, as verified in colorectal cancer lineages and reaffirmed in the study xenografted in mice, where the inhibitor suppressed the growth of colorectal tumor nodules [61]. The study focused on changes in the liver, as it is the first metastatic target of colorectal cancer, and cancer cells can induce the liver to secrete endocrine factors that facilitate tumor growth and transformation. Sporamin (infused intragastrically, 0.5 g/kg body weight/day for three weeks) was found to restore normal liver structure and downregulate the expression and secretion of β-catenin and VEGF in the liver, which subsequently inhibits the transcription of downstream genes involved in cancer progression and angiogenesis [61].

EcTI is cytotoxic to colorectal cancer cells (HCT116 and HT29), breast cancer cells (SkBr-3 and MCF-7), human leukemia cell lines (K562 and THP-1), and mouse fibrosarcoma (L929), without affecting normal human primary fibroblasts or human mesenchymal stem cells (hMSCs) [51,52,53,54]. Its action was investigated in more detail in the cellular events of a human gastric cancer line, Hs746T, and a nontumorigenic fibroblast cell line isolated from the human amniotic fluid [52]. EcTI treatment altered the morphology of Hs746T cells and blocked cell adhesion, migration, and invasion of gastric cancer cells, especially on the type I collagen matrix, impairing the formation of invadopodia. EcTI also decreased plasmin-induced pro-MMP-9 activation and activated pro-MMP-2 mediated by MT1-MMP [93], which is a unique characteristic of the inhibitor that appears to play an important role in its anticancer properties.

EcTI activity has also been studied in a glioblastoma (GBM) model, an aggressive brain tumor with poor overall survival [53]. Treatment of GBM remains a great challenge in oncology, and advanced cell therapies using normal human neural cells and hMSCs appear promising in preclinical experimental conditions, and are being studied as tools for cancer treatment because of their tropism to tumors and immunomodulatory ability. In GBM progression, proteases and their inhibitors are involved in the degradation of the extracellular matrix (ECM) as well as the regulation of other processes by proteolytic trimming and activation or inactivation of several signaling proteins. The effects of EcTI on GBM (U87 cells) and MSCs were compared with those on direct co-culture (MSC/U87) [53]. They showed that metabolic activity was less affected by EcTI in p53-wild type U87 cells than in MSC monoculture, although the metabolic rate of mixed co-culture was significantly reduced at lower EcTI concentrations. Under co-culture conditions, EcTI potentiated MSC-induced cell cycle arrest, probably due to a considerable increase in the expression of p53 and p21, and lower D1 level, but did not affect apoptosis. In addition, EcTI also enhanced Ca^2+^ signaling mediated via bradykinin receptor 2 in association with nitric oxide release, which highly impaired tumor cell proliferation and invasion. The mechanism did not seem to involve changes in cell adhesion, but rather the downregulation of β_1_ integrin signaling associated with p-FAK in U87 cells, both promoting inhibition of invasion. Finally, some cytokines were downregulated, suggesting that EcTI-mediated inhibition of signaling may be mediated by cytokines. Thus, in co-cultured MSC/U87 cells, EcTI impaired metabolic activity and proliferation, and reduced invasion, possibly through changes in cytokine secretion. In this context, plant-derived proteins potentiate the anticancer effects induced by MSCs, as demonstrated in the GBM U87 cell line. 

The effect of EcTI has also been evaluated in the triple-negative breast cancer cell line, MDA-MB-231. Decreased tumor cell viability and proliferation, as well as collagen I-mediated cell adhesion and the action of MMP-2 and MMP-9, enzymes that play an important role in the degradation of the extracellular matrix and promote tumor invasion and metastasis, have been reported. The inhibitor also reduced Akt and ERK levels, thereby enhancing the detrimental effects on cell viability and proliferation. NFκB, which is overexpressed in cancer and correlates with increased cell survival, was significantly reduced. Reduction in the levels of the proinflammatory cytokines IL-6 and IL-8 has also been reported, which, in addition to being expressed in cells that are resistant to several chemotherapeutic agents, are associated with NF-κB activation. In addition, there was an alteration in the integrin/FAK/SRC, BAX, and BCL-2 pathways, confirming the action of EcTI in carcinogenic processes [54].

Other protease inhibitors have also been studied for their roles in cancer progression. The effect of the recombinant form of the kallikrein inhibitor, rBbKI, on the viability of solid tumors was evaluated in the MKN-28 and Hs746T (gastric cancer), HCT116 (colorectal cancer), SkBr-3 and MCF-7 (breast cancer), and THP-1 and K562 (leukemia) cell lines. rBbKI inhibited cell viability in the Hs746T line more effectively than in nonmetastatic lines, such as MKN-28, HT-29, and HCT 116 [51]. In addition, the chimeric inhibitor rBbKIm [29], which has a peptide sequence containing the RGD motif of another *Bauhinia* inhibitor [14], has gained attention in tumor cell studies for its significant deleterious effect on prostate cancer. The potential mechanism involves the inhibition of tissue kallikreins, as these enzymes are important for the activation and development of this disease [55,100,101]. Ferreira et al. [29] demonstrated the differential action of rBbKIm on androgen-independent prostate cancer lines, PC3 and DU145, compared to normal amniotic fluid fibroblast cells and human endothelial cells (HUVECs). Treatment of the conditioned medium from the culture of DU145 and PC3 cells with rBbKIm for 48 h inhibited approximately 95% of the hydrolysis of HD-Pro-Phe-Arg-pNa by serine proteases of the kallikrein family. The importance of selective inhibitors of proteases with trypsin-like activity secreted by these cells may be reflected in cellular signaling events triggered by proteolysis, such as the activation of protease-activated receptors (PARs). PAR activation promotes cancer cell proliferation and invasion, and the release of angiogenic factors [101,102], and kallikreins are enzymes that cleave these receptors with a specificity similar to that of thrombin [103]. Thus, rBbKIm probably leads to the deficiency of these cellular events in prostate cancer via its direct inhibitory activity. In contrast, the RGD/RGE motif sequences contribute to recognizing integrins, regulating the invasive potential of metastatic tumor cells [104], and the increase in PAR1 level with the progression of prostate cancer [105]. Bilgin et al. [106] showed that the Kunitz-type inhibitor BbKI induced calcium release from the endoplasmic reticulum and consequently lowered the intracellular calcium concentration, which demonstrated a dose-dependent effect on the proliferation of HUVECs. In addition, BbKI was also found to cause hyperpolarization in the cell membrane, although the antiproliferative effect is independent of this property. Furthermore, rBbKI, a recombinant form of BbKI, affected L929 cell viability and cell adhesion to fibronectin, and it showed an interaction with the nucleus/cell surface of fibrosarcoma cells; however, rBbKI did not interfere with the cell cycle [55]. As highlighted previously, the trypsin inhibitor isolated from *Bauhinia rufa* (BrTI) has an atypical sequence containing the RGD and RGE motifs involved in cell adhesion [14]. The modified inhibitor rBbKIm, comprising RGD/RGE sequences [28], also interfered with the viability of prostate cancer cell lines DU145 and PC3, leading to cell death through the release of cytochrome c from, and caspase-3 activation in, the mitochondria [29].

BPLTI, isolated from seeds of *B. purpurea* L., is another protease inhibitor characterized from the genus *Bauhinia*. This inhibitor is a 20 kDa single polypeptide chain that shows relatively high thermal (0–60 °C) and pH (3–10) stability, and demonstrates antiproliferative and proapoptotic activities in human hepatocellular carcinoma HepG2 cells, as well as in MCF-7, CNE-1, and CNE-2 cells, but not in immortal human hepatic WRL 68 cells, L1210, HNE-2 tumor cells, or normal NP69 cells. Cell death tests revealed morphological changes in the nuclear structure of BPLTI-treated HepG2 cells, which reduced the number of cells and induced apoptosis proportional to the inhibitor dose. BPLTI also increased IL-1β transcript levels in a dose-dependent manner, and IL-2 and TNF-α levels at a specified time point. IL-1β and IL-2 levels increased from 4 to 16 h, while TNF-α levels began to decline after 4 h. However, BPLTI did not induce IFN-γ [40]. The selective action of plant protease inhibitors on tumor cells has also been demonstrated in a study by Shamsi et al. [41], in which a small trypsin and chymotrypsin Kunitz-type inhibitor (CCPI, MW~12 kDa) was characterized from pigeonpea (*Cajanus cajan* L.) seeds. CCPI acts on the adenocarcinoma human alveolar basal epithelial cell line A549, but does not affect the nontumor HEK cells. In another study, Karray et al. [44], investigated the highly stable PD inhibitor from *Conyza dioscoridis*, which showed cytotoxic effects on three human tumor cell lines (MDA-MB-231, HCT-116, and LoVo) without affecting HUVECs. Bonturi et al. [48] described CrataBL as an important bifunction protein (lectin and FXa inhibitor) in the glioblastoma tumor microenvironment, affecting U87 growth and invasion.

It is important to compare the effect of protease inhibitors with others in the Kunitz-type family to demonstrate that the effect is dependent on a specific protease. For example, the effect of rBbKIm was compared with the classical plant inhibitor of the Kunitz family, SbTI. In contrast to the inert effect of SbTI, rBbKIm was more selective, causing the death of prostate cancer cells [29]. This selectivity is promising for developing antitumor therapies, as cytotoxic drugs generally do not distinguish normal tissue from metastatic tissue, making it important to develop new compounds targeting proteins that are differentially expressed in cancer cells compared to normal adult tissue cells. The effect of protease inhibitors on the viability of nontumorigenic cells should therefore be analyzed to indicate selectivity for tumor cells. 

## 9. Concluding Remarks

We have gathered the main characteristics and effects of several protease inhibitors in this review. These inhibitors exert a wide range of effects, including antibacterial and insecticidal activity, inhibition of enzymes involved in the inflammation process, antithrombotic action, and the inhibition of tumor cell proliferation and invasion in vitro. Nevertheless, further studies are needed to enable a deeper understanding of their inhibitory mechanisms and facilitate their use as new therapeutic drugs.

## Figures and Tables

**Table 1:** Inhibitory Properties of some Plant Kunitz Inhibitors.

	Structural Characteristics			Serine Proteases Inhibition (K_i_ nM)									Cysteine Proteases Inhibition (K_i_ nM)	
Inhibitor	Cys Residues/S-S bonds/ Chains			Digestion Enzymes		Coagulation & Fibrinolysis Enzymes					Inflammatory Enzymes		Inflammatory Enzymes	
	Cys	S-S	Chains	Trypsin (Bovine)	Chymotrypsin (Bovine)	FXIIa	PKa (Human)	PKa (Rat)	FXa	Plasmin	HNE	Cat G	Cat L	Cat B
**EcTI**	4	2	2	0.9	1.1	82.0	55.0	n.d.	n.i.	9.4	55	n.d.	n.d.	n.d.
**BbKI**	1	0	1	0.6	2.7	n.d.	4.7	5.2	n.i.	33.0	n.i.	n.i.	n.i.	n.i.
**rBbKI**	1	0	1	20.0	n.i.	n.d.	2.0	n.d.	n.d.	33.0	n.i.	n.i.	n.i.	n.i.
**rBbKI-R64A**	1	0	1	25.0	n.i.	n.d.	98.0	n.d.	n.d.	2.6	n.i.	n.d.	n.i.	n.d.
**BrTI**	0	0	1	2.9	n.i.	n.d.	14	1.3	n.i.	n.i.	n.i.	n.i.	n.i.	n.i.
**BbCI**	0	0	1	n.i.	n.i.	n.i.	n.i.	n.i.	n.i.	n.i.	5.3	160.0	0.2	n.i.
**rBbCI**	0	0	1	n.i.	n.i.	n.d.	n.i.	n.d.	n.d.	n.i.	1.7	210.0	9.0	n.i.
**BuXI**	4	2	1	21.0	28.0	74.0	6.9	n.d.	14.0	76.0	n.i.	n.i.	n.d.	n.d.
**BvTI-C**	4	2	1	6.9	n.d.	n.d.	4.5	n.d.	n.i.	n.d.	n.d.	n.d.	n.d.	n.d.
**BvTI**	4	2	1	1.6	1.2	110	80.0	2.2	n.i.	2.9	n.i.	n.d.	n.d.	n.d.

n.i.—no detectable inhibition; n.d.—not determined.

**Table 2:** Functions of Some Kunitz Protease Inhibitors.

Plant Kunitz Inhibitor	Identification	Action
* Abelmoschus esculentus * lectin and Kunitz inhibitor	AEL	Toxicity against the fruit fly *Ceratitis capitata* and the nematodes *Meloidogyne incognita* and *M. javanica* [35]
* Adenanthera pavonina * Kunitz-type inhibitor	ApKTI	Insecticidal activity against *Plodia interpunctella* larvae [36] and reduces the survival of neonatal *Anticarsia gemmatalis* larvae [37]
* Alocasia macrorrhiza * Kunitz-type inhibitor	Alocasin	Increases the mortality rate of *Aedes aegypti* larvae and adult females [38]
* Bauhinia bauhinioides * cruzipain inhibitor and *B. mollis* elastase inhibitor	BbCI and BmEI	Anti-ulcer effects: regulation of pro-inflammatory processes and stress-induced gastric mucosal injuries [39]
*Bauhinia purpurea* L. trypsin inhibitor	BPLTI	Anti-proliferative and pro-apoptotic activities in HepG2, MCF-7, CNE-1, and CNE-2 cells [40]
* Cajanus cajan* L. Kunitz-type inhibitor	CCPI	Acts on the adenocarcinoma human alveolar basal epithelial cell line A549 [41]
* Cassia grandis * trypsin inhibitor	CGTI	Termiticidal activity against *Nasutitermes corniger* [42]
* Cassia leiandra * trypsin inhibitor	ClTI	Inhibits larvae development and delays adult emergence in *A. aegypti* causing mortality [43]
* Conyza dioscoridis * protease inhibitor	PDInhibitor	Cytotoxic effects on MDA-MB-231, HCT-116, and LoVo, without affecting HUVECs [44]
* Crataeva tapia * bark lectin	CrataBL	Controls the inflammatory response with a decrease in bronchoalveolar lavage, number of neutrophils, lymphocytes, eosinophils and mean alveolar diameter [45,46]; Impairs thrombus formation [47] and U87 cell microenvironment [48]
* Delonix regia * trypsin inhibitor and *Acacia schweinfurthii* trypsin inhibitor	DrTI and AsTI	Prolong the time for total carotid artery occlusion in mice and prevent arterial thrombus formation, without affecting bleeding time [49]
* Enterolobium contortisiliquum * trypsin inhibitor	EcTI	Induces the death of *Nasutitermes corniger* worker termites [50]; Cytotoxic to colorectal cancer cells (HCT116 and HT29), breast cancer cells (SkBr-3, MDA-MB-231 and MCF-7), human leukemia cell lines (K562 and THP-1), human gastric cancer (Hs746T), glioblastoma (U87) and mouse fibrosarcoma (L929), without affecting normal human primary fibroblasts and human mesenchymal stem cells (hMSCs) [51,52,53,54,55]
* Enterolobium timbouva * trypsin inhibitor	EtTI	Affects the integrity of the plasma membrane of yeasts, inducing the generation of intracellular ROS [56,57]
Recombinant *Hevea brasiliensis* amylase/subtilisin inhibitor	rHbASI	High activity against *Aspergillus oryzae* and inhibited mycelial growth of *Phytophthora palmivora* [34]
* Inga edulis * trypsin inhibitor	IeTI	Inhibits the pathogenic fungi *Candida tropicalis* and *Candida buinensis* [58]
* Inga laurina * trypsin inhibitor	ILTI	Inhibits trypsin midgut of lepidopteran pest: *S. frugiperda* [59]
* Inga vera * trypsin inhibitor	IVTI	Midgut inhibition of lepidopteran pests: *S. frugiperda*, C. *cephalonica*, *H. virescens*, and *H. zea* [60]
* Ipomoea potatoes * Kunitz-type trypsin inhibitor	Sporamin	Suppress the growth of colorectal tumor nodules in mice [61]
Kunitz-type trypsin inhibitor	KTI-A	Anti-proliferative activity in the melanoma cell line, B16F1 [62]
* Pseudostellaria heterophylla * trypsin inhibitor	PhTI	Potent activity against the pathogenic fungi *Candida gloeosporioides* and *Fusarium oxysporum* [63]
Recombinant *B. bauhinioides* kallikrein inhibitor	rBbKI	Termiticidal activity in workers termites (*Nasutitermes corniger*) [64]; Antithrombotic properties in venous and arterial thrombosis models [65]; Inhibits cell viability of MKN-28, HT-29, HCT 116, Hs746T [51] and L929 [55]
Recombinant *B. bauhinioides* kallikrein inhibitor modified	rBbKIm	Defense against *Callosobruchus maculatus* development [28]; Reduces cell viability in prostate cancer (DU145/PC3) leads to cell death [29]
* Rhynchosia sublobata * Kunitz isoinhibitor	RsKI	Actives against lepidopteran gut proteases: *Achaea Janata* and *Helicoverpa armigera* [66]
